# The deep sea biodiversity and conservation collection

**DOI:** 10.1038/s41598-024-77742-7

**Published:** 2024-11-11

**Authors:** Olga Sánchez, Sergio Stefanni, Punyasloke Bhadury

**Affiliations:** Bellaterra, Spain

**Keywords:** Biodiversity, Conservation biology

## Abstract

The deep sea, defined as ocean depths below 200 m, encompasses vast and largely unexplored habitats, such as abyssal plains, hydrothermal vents, cold seeps, and ocean trenches. This environment supports a remarkable diversity of life forms adapted to extreme conditions, including high pressure, low temperatures, and complete darkness. The *Deep Sea Biodiversity and Conservation* Collection highlights the importance of these ecosystems and the unique adaptations of the organisms inhabiting these extreme environments, ranging from invertebrates like corals and sponges to diverse microbial communities. The Collection includes studies on coral distribution and ecosystem services, trophic dynamics at cold-water coral reefs, and microbial diversity using metabarcoding and metagenomics. Notable findings include insights into hydrothermal vent communities, the role of chemosynthesis in sustaining deep-sea life, and the adaptation of deep-sea invertebrates to varying depths. These studies underscore the critical need for conservation strategies for these fragile and understudied oceanic ecosystems to ensure their sustainability.

The deep sea is broadly defined as the region of the ocean where light begins to fade, approximately below 200 m (656 feet), or marking the transition from the continental shelf to the continental slope. It harbors an extraordinary diversity of life forms that thrive in the most extreme and awe-inspiring environment on Earth. Encompassing vast and largely unexplored areas, the deep sea is home to an astonishing variety of organisms that have evolved to survive under intense pressure, perpetual darkness, and low temperatures. This includes abyssal plains, hydrothermal vents, cold seeps, and ocean trenches, each supporting unique communities with remarkable adaptations and resilience. Despite comprising the largest part of the world’s oceans, the deep sea remains a frontier for scientific research, offering valuable insights into the adaptability of life and underscoring the need for conservation to protect these fragile habitats.

The *Deep Sea Biodiversity and Conservation* Collection highlights the significance and potential of this largely unexplored ecosystem, which is home of communities of invertebrates such as corals, tubeworms, sponges, mollusks, crustaceans, as well as other organisms that rely on chemosynthesis to convert chemicals into energy. In addition to these macroscopic organisms, the deep sea hosts an incredible diversity of microscopic life forms, including bacteria, protists and viruses, which play crucial roles in nutrient cycling and contribute significantly to the overall functional biodiversity of these environments.

Among deep-sea benthic organisms, corals are widely distributed both geographically and vertically, occurring at depths ranging from the intertidal zone to over 6000 m. They are inextricably linked to the well being of human populations, supporting a large biomass and diversity while providing numerous ecosystem services, such as fisheries supplies, provision of calcareous and sand resources, and genetic reservoirs. Deep-sea corals have adapted to survive in these harsh environments, and several multidisciplinary studies included in this Collection provide a characterization of the habitats where cold-water corals thrive, helping to fill the knowledge gaps regarding their ecosystem function and value to human society. For example, Cordes et al.^1^ present the findings from recent surveys of the cold-water coral mound province on the Blake Plateau off the east coast of the United States, an area of intense human activity and home of some of the largest known deep-sea reef complexes. Their study comprehensively characterizes the Richardson Reef Complex, identifying the factors controlling cold-water coral reef distribution and the ecosystem services they provide to the region. They also incorporate these data into predictive models to estimate coral presence in unexplored parts of the region. On the other hand, Ramos et al*.*^2^ study uses mitochondrial genomes to provide insights into the evolutionary mechanisms that enable corals to inhabit such a wide range of depths and shed some light on their molecular response to the extreme conditions of the deep sea environment. Meanwhile, Vinya et al*.*^3^ use stable isotope analyses of carbon and nitrogen to unravel the trophic network of the Angolan cold-water coral reefs (southeast Atlantic), elucidating how organic carbon is transferred and cycled among different functional groups within these reef systems.

The Collection also includes various studies on hydrothermal vent ecosystems. For instance, Eilertsen et al*.*^4^ provide a comprehensive inventory of the fauna of the Loki’s Castle vent field on the Arctic Mid-Ocean Ridge, along with a preliminary food web analysis, revealing a community strongly influenced by chemosynthetic primary production. Similarly, Alfaro-Lucas et al.^5^ report a detailed characterization of the distribution, composition and trophic structure of the Capelinhos vent communities on the Mid-Atlantic Ridge, particularly those associated with mussel beds. They compare these findings with other Lucky Strike vent systems and other northern Mid-Atlantic Ridge vents, concluding that despite the chemical environment potential toxicity, the Capelinhos communities are as diverse as those at other vents, with methane oxidation playing a crucial role in their trophic dynamics.

Further contributions in the Collection focus on specific groups of deep-sea invertebrates. Ogiso et al*.*^6^ report on the peculiar adaptation of the marine worm, *Oligobrachia mashikoi*, which typically inhabits the deep sea and forms symbiotic association with sulphur bacteria. In a novel context, they report its behavioral response to temperature and light stimuli in shallow water environments.

The *Deep Sea Biodiversity and Conservation* Collection also explores the deep sea’s microbial diversity. For example, Peres et al*.*^7^ investigate prokaryotic communities in surface and subsurface deep sea sediments of the southwest Atlantic using metabarcoding and metagenomic approaches. Their findings revealed that the archaeal class Nitrososphaeria dominates in surface sediments, while the bacterial clade Dehalococcoidia is prevalent in the subsurface samples. Additionally, metagenomic analyses reveal the presence of the family CSP1-5, phylum Methylomirabilota, capable of methanol oxidation. On the other hand, Pernice et al.^8^ report the widespread occurrence of the fungus *Gjaerumia minor* (Basidiomycota), a plant parasite, in deep bathypelagic waters, especially in the Pacific Ocean. Their study suggests that this fungus may be transported to deep layers bound to particles, where it can grow and persist.

In conclusion, the deep sea is a realm of remarkable biodiversity and adaptability, home to life forms and ecosystems that challenge our understanding of biology and ecological function on Earth. Exploring and protecting this enigmatic world is not only a scientific imperative but also crucial for preserving the delicate balance of marine ecosystems and ensuring the long-term sustainability of life in the oceans and beyond for generations to come.
